# TNFAIP3 mutation may be associated with favorable overall survival for patients with T-cell lymphoma

**DOI:** 10.1186/s12935-021-02191-5

**Published:** 2021-09-15

**Authors:** Cunte Chen, Zheng Chen, Ling Huang, Lingling Zhou, Lihua Zhu, Sichu Liu, Gengxin Luo, Wenyu Li, Chengwu Zeng, Yangqiu Li

**Affiliations:** 1 Institute of Hematology, School of Medicine, Key Laboratory for Regenerative Medicine of Ministry of Education, Jinan University, Guangzhou, 510632 China; 2 Department of Lymphoma, Guangdong Provincial People’s Hospital, Guangdong Academy of Medical Sciences, School of Medicine, South China University of Technology, Guangzhou, China; 3 Department of Hematology, Nanfang Hospital, Southern Medical University, Guangzhou, China; 4 Department of Rheumatism and Immunology, First Affiliated Hospital, Jinan University, Guangzhou, China; 5 Department of Hematology, First Affiliated Hospital, Jinan University, Guangzhou, China

**Keywords:** *TNFAIP3* mutation, Mutation pattern, T-cell lymphoma, Prognosis

## Abstract

**Background:**

T-cell lymphoma (TCL) is highly aggressive and has a poor prognosis; thus, it is worth exploring biomarkers that may predict clinical outcomes and investigate their potential role in developing targeted therapies. In this study, we characterized the mutation pattern of tumor necrosis factor-alpha-inducing protein 3 (*TNFAIP3*) and its role in the prognosis of TCL patients.

**Methods:**

Coding sequence (CDS) mutations in *TNFAIP3* in TCL patients was explored using exome-sequencing data from 79 patients in our center (Guangdong Provincial People’s Hospital, GDPH) and 544 samples from the Catalogue of Somatic Mutations in Cancer (COSMIC) database. Additionally, non-CDS mutations in *TNFAIP3* in 41 TCL patients from our center (JNU) were investigated by polymerase chain reaction (PCR) and Sanger sequencing. Furthermore, non-CDS mutations in *TNFAIP3* in 47 TCL patients from Gene Expression Omnibus (GEO) dataset were explored.

**Results:**

In the COSMIC database, *TNFAIP3* mutations in TCL patients were located in the CDS, and the overall mutation frequency was 2.2%. However, *TNFAIP3* mutations were not detected in the CDS of any of the samples in our center’s datasets. Interestingly, non-CDS *TNFAIP3* mutations were found in 14.6% and 4.3% of TCL patients in the JNU and GSE15842 dataset, respectively. Importantly, there was a clear trend showing that TCL patients with a *TNFAIP3* mutation were associated with a longer 5-year restricted mean survival time (RMST) and favorable OS rate compared with those without a *TNFAIP3* mutation in the JNU dataset [hazard ratio (HR) = 0.29, 95% confidence interval (CI) 0.07 to 1.31, *P* = 0.089]. Furthermore, *TNFAIP3* mutations significantly correlated with T-cell large granular lymphocytic leukemia (T-LGLL) with a favorable prognosis in the JNU dataset (*P* = 0.002). Notably, the different mutation patterns of *TNFAIP3* when comparing our center and the COSMIC datasets might be due to different ethnic and genetic backgrounds.

**Conclusions:**

To the best of our knowledge, we for the first time describe that *TNFAIP3* mutations in non-CDS regions are associated with favorable OS for TCL patients, which might be a potential biomarker for the prognostic stratification of Chinese TCL patients.

**Supplementary Information:**

The online version contains supplementary material available at 10.1186/s12935-021-02191-5.

## Introduction

T-cell lymphoma (TCL) originates from lymphoblasts or mature T cells. This disease accounts for only 10–15% of non-Hodgkin’s lymphoma and can be further subdivided into many relatively rare subtypes [1–5]. Patients with TCL tend to have a poor prognosis when compared to those with B-cell lymphoma [6–8]. Implementation of a risk stratification model based on the international prognosis index (IPI) has made significant progress in predicting the prognosis of TCL patients, and this refined stratification can influence clinical decision-making, thereby improving the prognosis of patients [9, 10]. However, the current risk stratification based on IPI cannot accurately predict prognoses for all TCL patients [9, 11]. Therefore, it is worth exploring novel biomarkers that further improve risk stratification for TCL.

The intracellular ubiquitin editing protein tumor necrosis factor alpha-induced protein 3 (*TNFAIP3*), also known as A20, negatively regulates the activity of NF-κB in a variety of pathways through tumor necrosis factor and Toll-like receptors [12–14]. The *TNFAIP3* gene locus is located in chromosome band 6q23, and its deletion frequently occurs in B-cell lymphomas, particularly extranodal marginal zone B cell lymphoma and diffuse large B-cell lymphoma [15–17]. Additionally, a few previous studies have revealed that *TNFAIP3* deletion is frequently found in cutaneous T-cell lymphoma (CTCL) and NK-T cell lymphoma (NKTCL) [15, 18, 19]. Moreover, the prognostic value of *TNFAIP3* deletion in NKTCL patients has been investigated, but the results are contradictory [15, 19]. In our previous study, mutations in the non-coding sequence (non-CDS) region of *TNFAIP3* were also found in T-cell hematological malignancies [20, 21]. However, comprehensive assessment of the mutation pattern and prognostic importance of *TNFAIP3* in Chinese TCL patients in multiple large datasets remains lacking.

In this study, we investigated the *TNFAIP3* mutation pattern and its prognostic value for TCL patients from Jinan University (JNU), Guangdong Provincial People’s Hospital (GDPH), Catalogue of Somatic Mutations in Cancer (COSMIC), and Gene Expression Omnibus (GEO) datasets.

## Materials and methods

### GDPH and JNU datasets

**Fig. 1:**
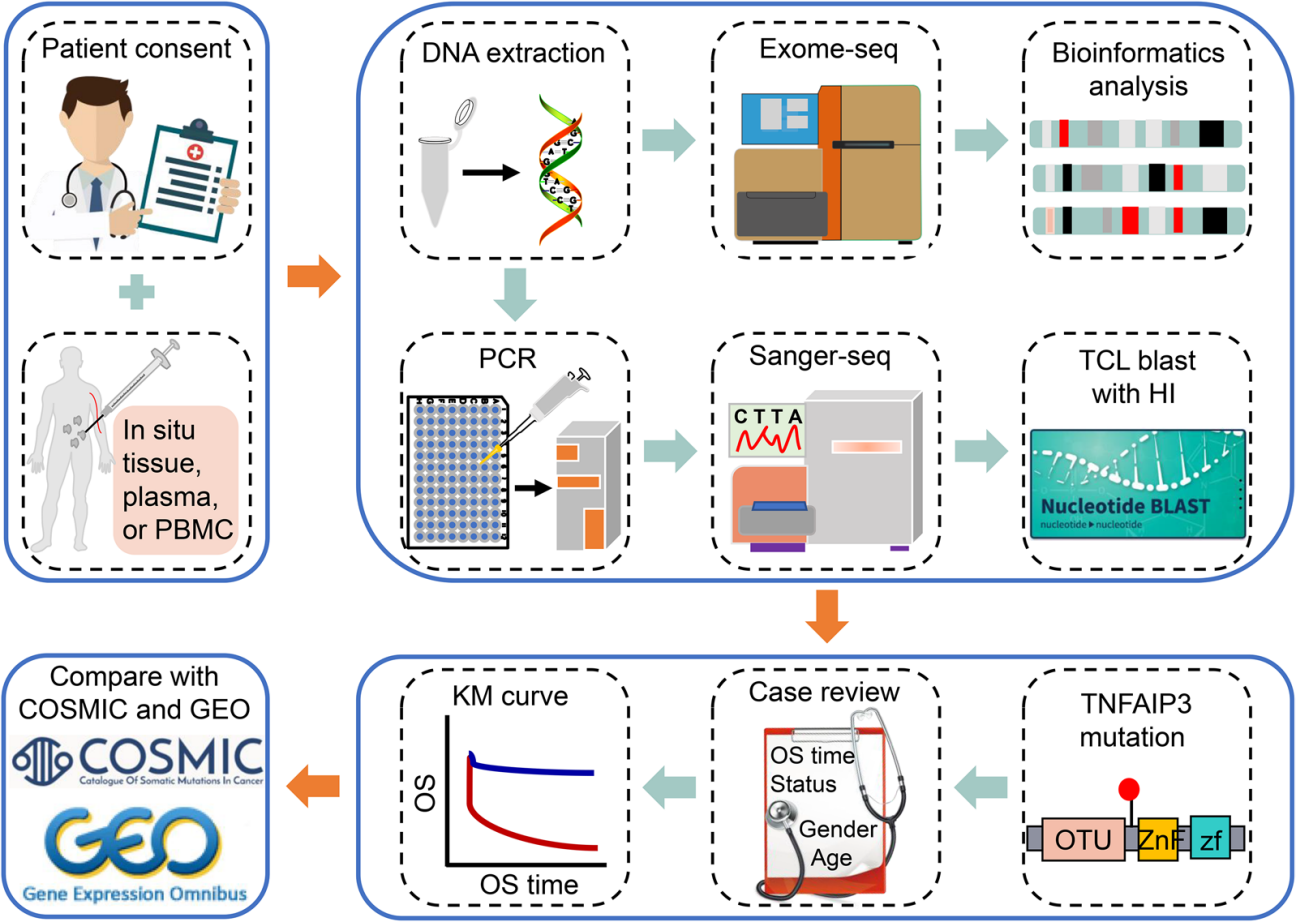
Schematic diagram of the study. All patients provided informed consent to collecting T-cell lymphoma (TCL) in situ tumor tissue, plasma, or peripheral blood mononuclear cells (PBMCs) for deoxyribonucleic acid (DNA) extraction. Then, the prepared DNA was subjected to exome sequencing for bioinformatics analysis or polymerase chain reaction (PCR) and Sanger sequencing for comparison with healthy individual (HI) sequences, which identified the variant sites and types of *TNFAIP3* mutations. Finally, the clinical information of the TCL patients was collected for prognostic analysis, and the *TNFAIP3* mutation data from Jinan University (JNU) and Guangdong Provincial People’s Hospital (GDPH) were compared to those in the Catalogue of Somatic Mutations in Cancer (COSMIC)  and Gene Expression Omnibus (GEO) databases

A total of 79 tumor biopsies and whole blood samples were collected from de novo TCL patients at GDPH between February 27, 2015 and December 17, 2020, and these samples were used for exome sequencing. In the GDPH dataset, the TCL subtypes accounting for greater than 10% of the samples were as follows: T-lymphoblastic lymphoma (T-LBL) (22.8%), angioimmunoblastic T-cell lymphoma (AITL) (22.8%), peripheral T-cell lymphoma, not otherwise specified (PTCL-NOS) (22.8%), and NKTCL (19.0%). Moreover, peripheral blood mononuclear cells (PBMCs) from 41 de novo TCL patients from JNU between October 19, 2007 and January 27, 2016 and 30 healthy individuals (HIs) were obtained for PCR and Sanger sequencing [20, 21] (Fig. 1). In the JNU dataset, TCL subtypes accounting for greater than 10% of the samples were as follows: NKTCL (36.6%), T-LBL (22.0%), AITL (12.2%), and PTCL-NOS (12.2%). Corresponding clinical information, including gender, age, status, and overall survival (OS) time, was also collected (Table 1). The patients in the JNU and GDPH datasets are all from China (100%). TCL subtypes were determined according to the 2008 World Health Organization (WHO) classification of tumors of hematopoietic and lymphoid tissues [22]. The date of the last follow-up was May 1, 2021, and the median follow-up time for the surviving TCL patients in the JNU dataset was 23.37 months (range: 0.23–157.83 months). OS was defined as the span from diagnosis to death from any cause or last follow-up. All procedures involving human participants were performed according to the principles of the Declaration of Helsinki and approved by the Ethics Committee of the First Affiliated Hospital of Jinan University. All patients provided written informed consent.

**Table 1:** Clinical characteristics of TCL patients

Variables	JNU dataset	GDPH dataset	COSMIC dataset	GSE15842 dataset
Number	41	79	544	47
Gender, n (%)			–	–
Female	19 (46.3)	30 (38.0)	–	–
Male	22 (53.7)	49 (62.0)	–	–
Age, y, mean ± SD	48 ± 19	46 ± 19	–	–
Subtype, n (%)^a^				
AITL	**5 (12.2)**	**18 (22.8)**	**101 (18.6)**	0 (0.0)
ALCL	1 (2.4)	6 (7.6)	19 (3.5)	0 (0.0)
ATLL	0 (0.0)	0 (0.0)	**292 (53.7)**	0 (0.0)
CTCL	1 (2.4)	1 (1.3)	**73 (13.4)**	0 (0.0)
EATL	1 (2.4)	0 (0.0)	0 (0.0)	0 (0.0)
HSTCL	0 (0.0)	1 (1.3)	3 (0.6)	0 (0.0)
MEITL	0 (0.0)	1 (1.3)	0 (0.0)	0 (0.0)
NKTCL	**15 (36.6)**	**15 (19.0)**	7 (1.3)	0 (0.0)
PTCL-NOS	**5 (12.2)**	**18 (22.8)**	48 (8.8)	**47 (100%)**
SPTCL	1 (2.4)	1 (1.3)	0 (0.0)	0 (0.0)
T-LBL	**9 (22.0)**	**18 (22.8)**	0 (0.0)	0 (0.0)
T-LGLL	3 (7.3)	0 (0.0)	1 (0.2)	0 (0.0)
Country, n (%)^a^				
China	41 (100)	79 (100)	0 (0.0)	0 (0.0)
USA	0 (0.0)	0 (0.0)	237 (43.6)	0 (0.0)
Japan	0 (0.0)	0 (0.0)	291 (53.5)	0 (0.0)
Australia	0 (0.0)	0 (0.0)	9 (1.7)	0 (0.0)
Italy	0 (0.0)	0 (0.0)	5 (0.9)	0 (0.0)
Germany	0 (0.0)	0 (0.0)	0 (0.0)	47 (100%)
Unknown	0 (0.0)	0 (0.0)	4 (0.7)	0 (0.0)
Detection method	PCR + sanger sequencing	Exome sequencing	–	SNP array
Covered area	Non-CDS and CDS	CDS	CDS	–
Follow up, m, median (range)	23.37 (0.23 to 157.83)	–	–	–
Status (alive/dead), n	22/19	–	–	–

The bold values indicated that the proportion of TCL subtypes accounting for more than 10% of the sample

*AITL* angioimmunoblastic T-cell lymphoma, *ALCL* anaplastic large cell lymphoma, *ATLL* adult T-cell lymphoma-leukemia, *CDS* coding sequence, *COSMIC* catalogue of somatic mutations in cancer, *CTCL* cutaneous T-cell lymphoma, *EATL* enteropathy*-*associated T*-*cell lymphoma, *GDPH* Guangdong Provincial People’s Hospital, *HSTCL* hepatosplenic T-cell lymphoma, *JNU* Jinan University, *MEITL* monomorphic epitheliotropic intestinal T-cell lymphoma, *NGS* next-generation sequencing, *NKTCL* NK-T-cell lymphoma, *PCR* polymerase chain reaction, *PTCL-NOS* peripheral T-cell lymphoma, not otherwise specified, *SNP* single nucleotide polymorphism, *SPTCL* subcutaneous panniculitis-like T-cell lymphoma, *T-LBL* T-lymphoblastic lymphoma, *T-LGLL* T-cell large granular lymphocytic leukemia

^a^Due to rounding, not all percentages total 100%

### COSMIC and GSE15842 datasets

Mutation data from 544 TCL patients from 12 clinical centers were downloaded from the COSMIC database (https://cancer.sanger.ac.uk/cosmic). Furthermore, single nucleotide polymorphism (SNP) data of 47 German patients with PTCL-NOS in GSE15842 datasets were downloaded from the GEO database (https://www.ncbi.nlm.nih.gov/geo/). In addition, the countries of origin for the TCL patients as well as TCL subtypes were obtained (Table 1). The TCL subtypes accounting for greater than 10% of the COSMIC dataset included adult T-cell lymphoma-leukemia (ATLL) (53.7%), AITL (18.6%), and CTCL (13.4%). Notably, the TCL patients in the COSMIC dataset are from the USA (43.6%), Japan (53.5%), Australia (1.7%), Italy (0.9%), and unknown (0.7%). The COSMIC and GEO databases are publicly available; thus, approval from the local ethics committee was not required.

### Exome sequencing

Tumor genomic DNA was extracted from tumor biopsies and plasma for library construction, exome sequencing, sequence alignment and processing, and single nucleotide variation (SNV), insertion and deletion (indel), and copy number variation (CNV) detection was performed (Fig. 1). The detailed exome sequencing process was described previously [23].

### Polymerase chain reaction (PCR) and Sanger sequencing

The promoter, exons 2–9, and 3′ UTR of the *TNFAIP3* gene were amplified by 16 primer pairs, and the primer sequences are shown in Additional file 1: Table S1. PCR was performed as previously described [20, 21]. After the PCR products were Sanger sequenced [20, 21], TCL and HI sequences were compared using BLAST software (http://blast.ncbi.nlm.nih.gov/Blast.cgi) to the determine variant sites and types of *TNFAIP3* mutations.

### Statistical analysis

All statistical analysis was conducted using R (version 4.0.2, https://www.r-project.org/) [24]. Kaplan–Meier curves were plotted by the R package “survival’’, which was compared using the log-rank test [25, 26]. The R package “survRM2’’ was also used to determine the restricted mean survival time (RMST) [27]. Differences in two groups of qualitative variables were compared by the Fisher’s exact test. A two-tailed *P* < 0.05 and a *P* < 0.1 were considered statistically significant and a clear trend, respectively.

## Results

### Mutation pattern of *TNFAIP3* in TCL

**Fig. 2:**
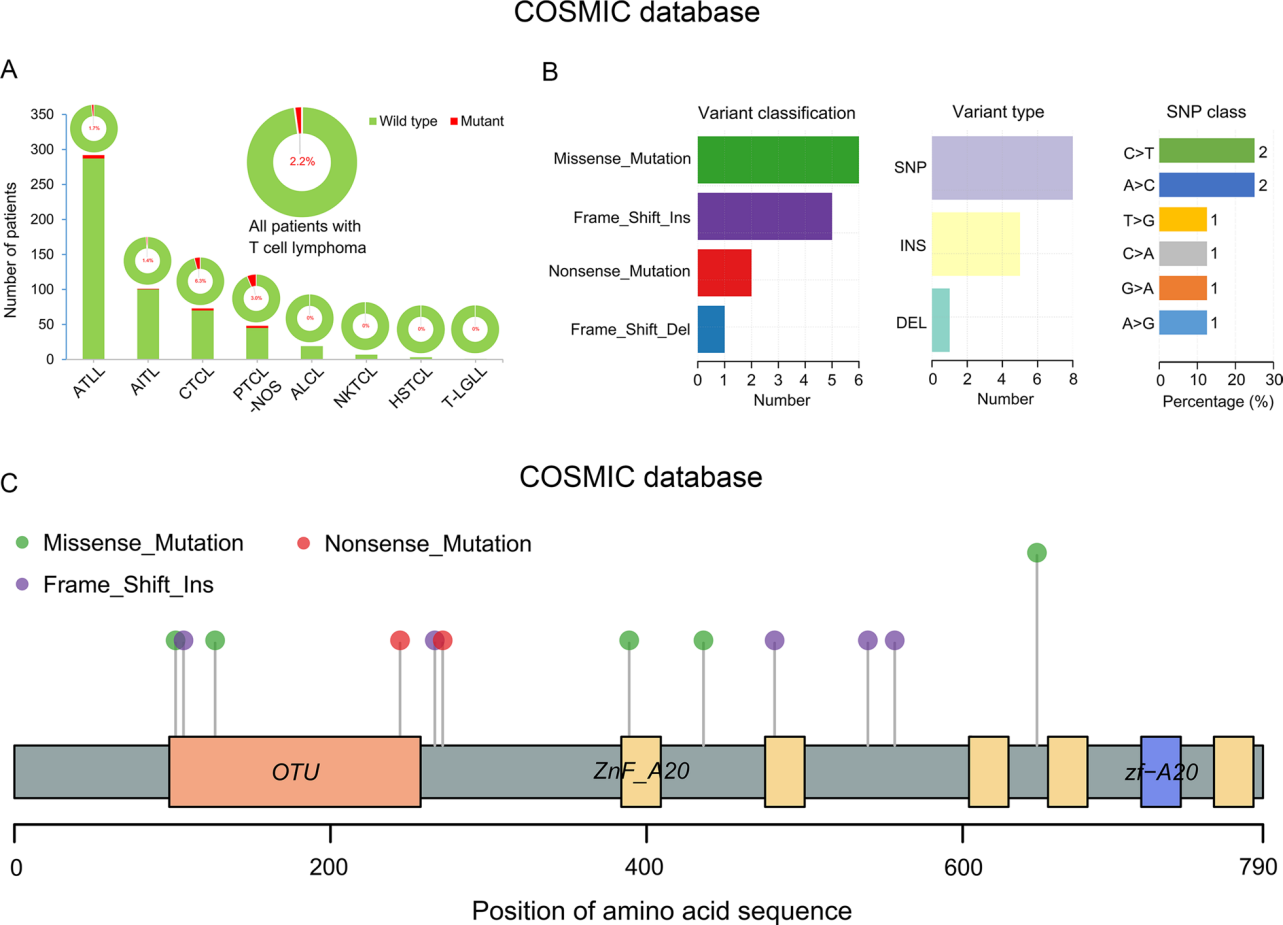
The variant sites, types of *TNFAIP3* mutations, and percentage of mutations in the COSMIC dataset. **A**, **B** The percentage of *TNFAIP3* mutations in TCL patients (**A**), and variant classification, variant type, and single nucleotide polymorphism (SNP) class (**B**) in the COSMIC database. **C** The package “maftools” in R (version 4.0.2, https://www.r-project.org/) was used to obtain the variant sites in *TNFAIP3* in the COSMIC database

**Fig. 3:**
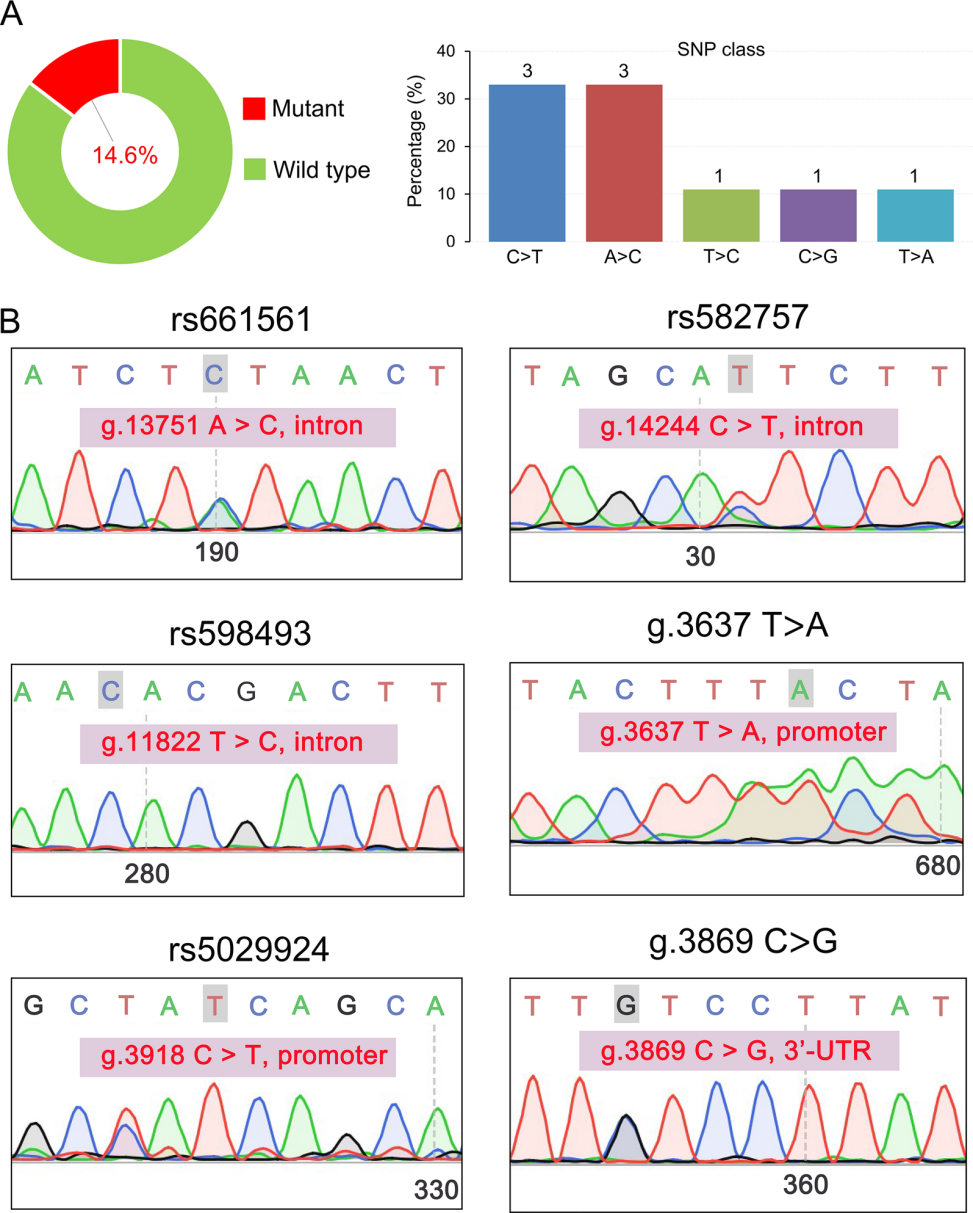
The *TNFAIP3* mutation pattern in the JNU dataset. **A** Percentage of *TNFAIP3* mutations (left panel) and SNP class (right panel) in TCL patients from the JNU dataset. **B** Representative variant sites in the *TNFAIP3* introns, promoter, and 3′ untranslated region (3′UTR) were obtained from the SNP database (https://www.ncbi.nlm.nih.gov/snp/)

In COSMIC samples, all of the *TNFAIP3* mutations are found in the CDS region with an overall mutation frequency of 2.2% (12/544) in TCL (Table 2; Fig. 2A). The CTCL mutation frequency was relatively high, reaching 6.3%, and no *TNFAIP3* mutations were found in anaplastic large cell lymphoma (ALCL), NKTCL, hepatosplenic T-cell lymphoma (HSTCL), or large granular T lymphocyte leukemia (T-LGLL) (Fig. 2A). We further analyzed the types of mutations and found that missense, frameshift insertions, nonsense, and frameshift deletions mainly occurred in the *TNFAIP3* gene, and the single base substitutions C>T (positions g.811 and g.1166) and A>C (position g.1939) were significantly prominent (Fig. 2B, C). However, in samples obtained from our center (GDPH and JNU datasets), there were no mutations detected in the *TNFAIP3* CDS region by high-throughput sequencing, PCR, or Sanger sequencing. However, we did detect mutations in non-CDS regions in TCL samples in the JNU dataset by both PCR and Sanger sequencing. Interestingly, we found that 14.6% (6/41) of TCL patients had a *TNFAIP3* mutation in the non-CDS region (Table 2; Fig. 3A, left panel). Among these mutations, C>T alterations (positions g.3918 and g.14244) and A>C (position g.13751) were the most common in TCL (Fig. 3A, right panel and 3B). Further subgroup analysis revealed that the TCL subtypes with a *TNFAIP3* mutation included 3/6 T-LGLL, 2/6 T-LBL, and 1/6 CTCL (Table 2). Notably, 4.3% (2/47) of PTCL-NOS patients had a *TNFAIP3* mutation in the non-CDS region in the GSE15842 dataset (Table 2).

**Table 2:** Clinical characteristics of T-cell lymphoma patients with a *TNFAIP3* mutation

Sample ID	Histological subtype	Age, y	Gender	Country	Mutation sites	*TNFAIP3* mutation	OS time (months)	Clinical outcome
COSMIC dataset								
Patient 1	PTCL-NOS	< 60	Female	USA	CDS	g.305 A>G	–	Not relapse
Patient 2	PTCL-NOS	≥ 60	Female	USA	CDS	g.1939 A>C	–	Not relapse
Patient 3	PTCL-NOS	–	–	USA	CDS	g.1939 A>C	–	Not relapse
Patient 4	CTCL	≥ 60	Male	USA	CDS	g.1307G>A, g.796dup, and g.732 C>A	–	Alive
Patient 5	AITL	–	Female	USA	CDS	g.811 C>T	–	Alive
Patient 6	CTCL	67	Male	USA	CDS	g.-15-1829_-15-1814del	–	–
Patient 7	ATLL	56	Male	Japan	CDS	g.318dup	–	–
Patient 8	CTCL	–	Female	USA	CDS	g.380T>G	–	–
Patient 9	ATLL	–	–	Japan	CDS	g.1616_1617insT	–	–
Patient 10	ATLL	–	–	Japan	CDS	g.1439_1440dup	–	–
Patient 11	ATLL	79	Female	Japan	CDS	g.1667dup	–	–
Patient 12	ATLL	–	–	Japan	CDS	g.1166 C>T	–	–
GSE15842 dataset								
Patient 13	PTCL-NOS	–	–	Germany	Intron	rs582757	–	–
Patient 14	PTCL-NOS	–	–	Germany	Intron	rs582757	–	–
JNU dataset								
Patient 15	T-LBL	69	Female	China	Promoter	g.3918 C>T	98.9	Death
Patient 16	CTCL	36	Male	China	Promoter	g.3918 C>T and g.3637 T>A	58.8	Death
Patient 17	T-LBL	19	Male	China	3’UTR	g.3869 C>G	94.3	Alive
Patient 18	T-LGLL	27	Male	China	Intron	g.13751 A>C	97.1	Alive
Patient 19	T-LGLL	40	Female	China	Intron	g.13751 A>C	118.5	Alive
Patient 20	T-LGLL	50	Female	China	Intron	g.13751 A>C, g.14244 C>T, and g.11822T>C	102.4	Alive

*AITL* angioimmunoblastic T-cell lymphoma, *ATLL* adult T-cell lymphoma-leukemia, *CDS* coding sequence, *CTCL* cutaneous T-cell lymphoma, *JNU* Jinan University, *OS* overall survival, *PTCL-NOS* peripheral T-cell lymphoma, not otherwise specified, *T-LBL* T-lymphoblastic lymphoma, *T-LGLL* T-cell large granular lymphocytic leukemia, *UTR* untranslated region

### *TNFAIP3* mutation correlates with favorable OS for TCL patients

**Fig. 4:**
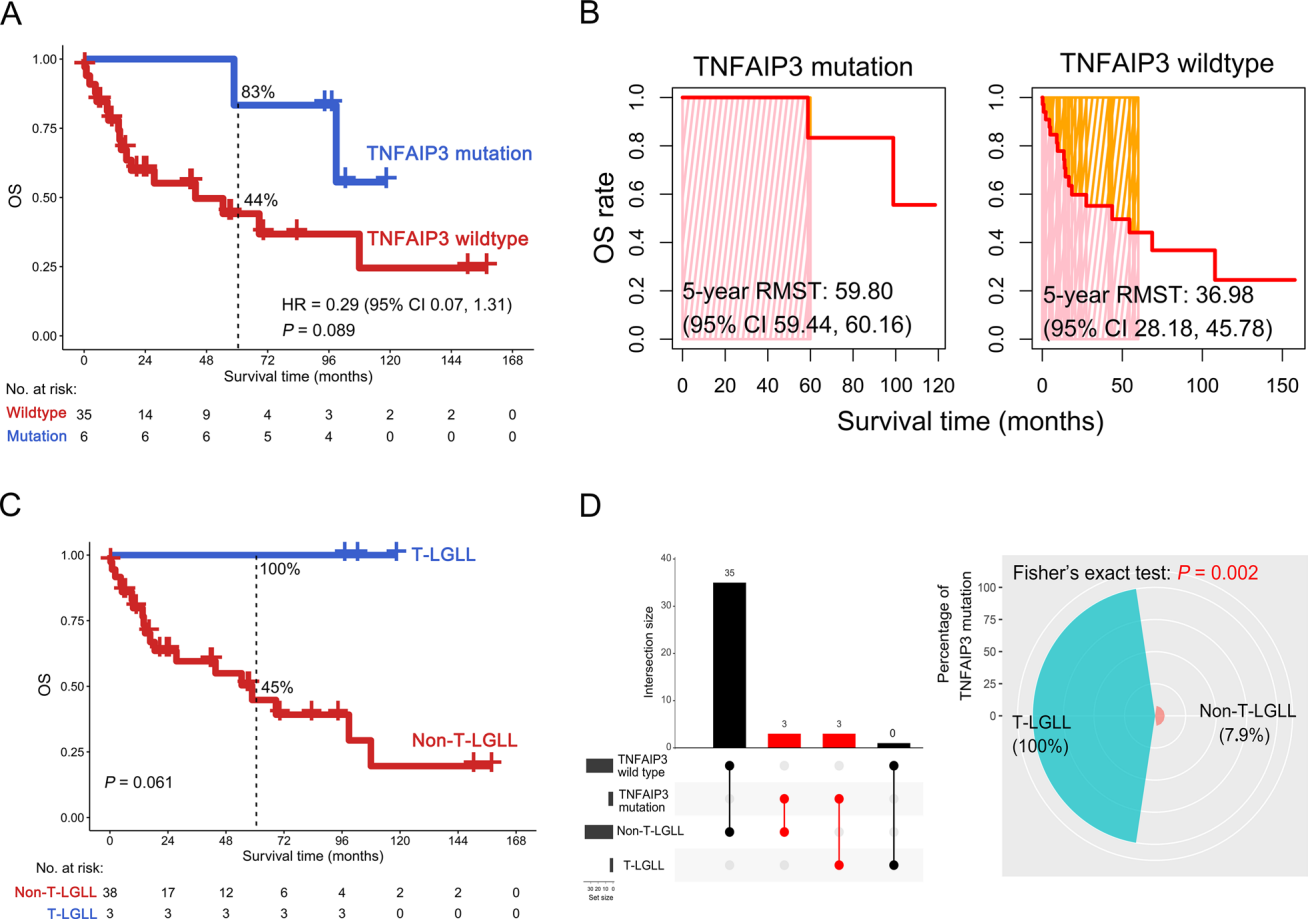
Overall survival (OS) analysis of *TNFAIP3* mutaions in TCL patients in the JNU dataset. **A**, **B** Kaplan–Meier curve (**A**) and 5-year restricted mean survival time (RMST) (**B**) based on the status of *TNFAIP3* mutations are shown. **C** Overall survival (OS) analysis of T-cell large granular lymphocytic leukemia (T-LGLL) and non-T-LGLL patients. **D** The number (left panel) and percentage (right panel) of *TNFAIP3* mutations in T-LGLL and non-T-LGLL patients

To further investigate the relationship between *TNFAIP3* mutation and OS for TCL patients, we conducted a prognostic analysis. Interestingly, there was a clear trend where TCL patients harboring a *TNFAIP3* mutation in non-CDS regions in our center (JNU dataset) were associated with favorable OS, although the data were not yet statistically significant at this point [hazard ratio (HR) = 0.29; 95% confidence interval (CI): 0.07 to 1.31; 5-year OS rate: 83% vs. 44%; *P* = 0.089] (Fig. 4A). Moreover, RMST was used to evaluate the performance of the Kaplan-Meier curve. As shown in Fig. 4B, the 5-year RMST for TCL patients with or without a *TNFAIP3* mutation in the JNU dataset was 59.80 (95% CI 59.44 to 60.16) and 36.98 (95% CI 28.18 to 45.78) months, respectively. To clarify the relationship between *TNFAIP3* mutation and TCL subtypes, we next conducted a correlation analysis. As shown in Fig. 4C, there was a clear trend indicating that T-LGLL patients were associated with favorable OS compared to non-T-LGLL patients (5-year OS rate: 100% vs. 45%; *P* = 0.061), although the data were not yet statistically significant at this point. Interestingly, *TNFAIP3* mutation was positively correlated with T-LGLL (*P* = 0.002, Fig. 4D).

## Discussion

*TNFAIP3* is a well-known negative regulator of the NF-κB signaling pathway, and mutations in this gene are usually observed in cancer, including hematological malignancies, which affect the clinical outcome of patients [13, 28–30]. Additionally, the mutation of *TNFAIP3* has been widely studied in B-cell lymphoma [13, 16, 31], but its mutation pattern and impact on the prognosis of TCL patients are little known [15]. Because there are few studies regarding the alteration of *TNFAIP3* in T cell malignancies in China. In this study, we firstly focused on comparing the differences in CDS mutations of *TNFAIP3* between China and other countries. Interestingly, we observed this different pattern of *TNFAIP3* mutation in CDS in Chinese TCL patients. We suggested that it may be due to the differences in population distribution characteristics. This suggestion is also based on our previous finding on the different SNP distribution of *TNFAIP3* in CDS in patients with rheumatoid arthritis in comparison with patients from other countries [32]. Further confirmation for this alteration will be done in another cohort of T cell lymphoma samples from China. Notably, 2.2% of TCL patients had mutations in the CDS region of *TNFAIP3* in the COSMIC dataset, but none were found in our clinical center’s datasets. Interestingly, we further examined the non-CDS region of *TNFAIP3* and found that 14.6% of TCL patients had mutations in the JNU dataset. We did try to find the feature of *TNFAIP3* mutation in the non-CDS region from different datasets. The only one dataset with SNP data of *TNFAIP3* is the GSE15842 dataset. 4.3% of TCL patients had a *TNFAIP3* mutation in the non-CDS region. However, there is no prognostic information in the GSE15842 dataset for us to analyze the correlation between the non-CDS mutation of *TNFAIP3* and the prognosis of TCL patients. There was a report describing bi- and monoallelic deletions of *TNFAIP3* in a high proportion of CTCL patients [18]. Importantly, our work uncovered that 6.3% of CTCL patients have *TNFAIP3* mutations, and one of which was a deletion mutation found in the COSMIC database. Moreover, although a few studies have shown that *TNFAIP3* deletion is detected in 18–35% of extranodal NKTCL (ENKTCL) patients [15, 19], no mutations in *TNFAIP3* in this subtype were found in this study. Furthermore, single base-pair mutations were detected rather than deletions in the 3′UTR, promoter, and intron regions using PCR, Sanger, and exome sequencing in our clinical center’s datasets. Finally, *TNFAIP3* mutation significantly correlated with T-LGLL. This study identified the distribution characteristics of TNFAIP3 mutations in TCL subtypes. Recently, researchers have explored the prognostic value of *TNFAIP3* deletion in patients with ENKTCL, but inconsistent results have been found. Ahn et al. reported that *TNFAIP3* deletions were associated with poor progression-free survival (PFS) in ENKTCL patients, while Liu et al. suggested that heterozygous deletions in *TNFAIP3* predicted favorable OS in ENKTCL [15, 19]. In previous reports, *TNFAIP3* negatively regulates the activity of NF-κB by up-regulating the expression level to inhibit tumor development. While, *TNFAIP3* was also thought to be an oncogene in numbers of solid tumors such as ductal carcinoma in situ, gastric carcinoma, basal-like breast cancer [33–35]. Moreover, TNFAIP3 promotes CD4+ T cell survival by restricting ubiquitination of the mTOR (mechanistic target of rapamycin kinase) complex and increased autophagy [36]. In this study, we found that TCL patients with *TNFAIP3* mutations were associated with favorable OS. However, we had not done the experiment to characterize the role of the mutated *TNFAIP3* in TCL cells. Therefore, we can only report the first finding which may be a novel biomarker for predicting the clinical outcome of TCL. And we will further explore the role of such mutations in malignant T cells to characterize their function and the related mechanism of the patients carrying non-CDS mutation of *TNFAIP3* with favorable OS. It is known that T-LGLL patients have a favorable prognosis with a 30-year OS rate of up to 70% [37, 38], which is consistent with this study. Interestingly, the results of this study indicated that mutant-*TNFAIP3* significantly correlates with T-LGLL. Indeed, it would be perfect, that we could show the validation results either from the database or from another cohort of clinical samples. We did try to obtain another dataset from the publicly available database to validate our results. Unfortunately, there is no dataset containing both prognosis and *TNFAIP3* mutation information for us to further analyze and validate. Thus, on the other hand, our data may be provided the first information of *TNFAIP3* mutation in non-CDS linking to prognosis of TCL, which may be a potential biomarker for predict the prognosis of patients with TCL. Therefore, a larger cohort samples are needed to further validate the relationship between *TNFAIP3* mutation and favorable OS in the future.

The difference between the *TNFAIP3* mutation pattern in the COSMIC dataset and our clinical center’s datasets may be due to the following reasons. First, the population distribution characteristics of TCL subtypes will lead to differences in the *TNFAIP3* mutation detection rate. In the GDPH and JNU datasets, NKTCL, T-LBL, AITL, and PTCL-NOS comprised the TCL subsets with the highest percentages, while in the COSMIC dataset, they were ATLL, AITL, and CTCL. Second, the TCL patients in the three datasets come from different countries, which may be a reason why the *TNFAIP3* mutation patterns were inconsistent. The TCL patients in the JNU and GDPH datasets all come from China, while in the COSMIC dataset, they come from the USA, Japan, Australia, and Italy. Therefore, it is necessary to further confirm the role of mutant *TNFAIP3* in the evaluation of OS for TCL patients according to the distribution characteristics of TCL subtypes in different countries.

## Conclusions

We for the first time demonstrate a low mutation frequency of *TNFAIP3* in TCL, and the mutations were primarily located in the non-CDS region in Chinese patients. Notably, mutant *TNFAIP3* might be a predictor of favorable OS in TCL patients, which might complement current risk stratification of Chinese TCL patients.

## Supplementary Information

**Additional file 1: Table S1.** PCR primers for *TNFAIP3*.

## Data Availability

The COSMIC data used in this study were acquired from the COSMIC database (https://cancer.sanger.ac.uk/cosmic). The datasets used and/or analyzed during the current study are available from the corresponding author on reasonable request.

## Abbreviations

AITL: Angioimmunoblastic T-cell lymphoma
ALCL: Anaplastic large cell lymphoma
ATLL: Adult T-cell lymphoma-leukemia
CDS: Coding sequence
CI: Confidence interval
COSMIC: Catalogue of somatic mutations in cancer
CTCL: Cutaneous T-cell lymphoma
EATL: Enteropathy*-*associated T*-*cell lymphoma
GDPH: Guangdong Provincial People’s Hospital
HI: Healthy individual
HSTCL: Hepatosplenic T-cell lymphoma
HR: Hazard ratio
Indel: Insertion and deletion
IPI: International prognosis index
JUN: Jinan University
NGS: Next-generation sequencing
NKTCL: NK-T-cell lymphoma
OS: Overall survival
PCR: Polymerase chain reaction
PBMC: Peripheral blood mononuclear cell
PTCL-NOS: Peripheral T-cell lymphoma, not otherwise specified
RMST: Restricted mean survival time
SNV: Single nucleotide variation
SPTCL: Subcutaneous panniculitis-like T-cell lymphoma
T-LGLL: T-cell large granular lymphocytic leukemia
TCL: T-cell lymphoma
*TNFAIP3*: Tumor necrosis factor alpha-induced protein 3
