# Supplementary material for: TNFAIP3 mutation may be associated with favorable overall survival for patients with T-cell lymphoma
Source: Cancer Cell Int. 2021 Sep 15;21:490. doi: 10.1186/s12935-021-02191-5 (PMC8444556; doi:10.1186/s12935-021-02191-5)
Supplement: Supplementary file 1 — Additional file 1: Table S1. PCR primers for TNFAIP3. [file 12935_2021_2191_MOESM1_ESM.doc]

**Table S1.** PCR primers for *TNFAIP3*.

| Targets | Primers | Sequence (5′–3′) |
| --- | --- | --- |
| Exon 2 | exon2 (F) | GGAGTCGTATTAAAGTCAGGCTAA |
|  | exon2 (R) | GGCAAAAGAAACACAACAGAAC |
| Exon 3 | exon3 (F) | TTGCTGGGTCTTACATGCAG |
|  | exon3 (R) | CCCACCATGGAGCTCTGTTA |
| Exon 4 | exon4 (F) | GGGAGTACAGGATACATTCAAGC |
|  | exon4 (R) | GCTGAAAGCATTTAAGTACAGATCC |
| Exon 5 | exon5 (F) | ACCTAAGGGCCTCATTTTCC |
|  | exon5 (R) | AGCAAAAAGGAAAACCCTGA |
| Exon 6 | exon6 (F) | TGAGATCTACTTACCTATGGCCTTG |
|  | exon6 (R) | CAGATGACACAGGAGAGAGCTG |
| Part of exon 7 | exon7-1 (F) | GGTTCTACAATTCTTGCCATAATCC |
|  | exon7-1 (R) | CAAGTGCCTTGTGTGGTCTG |
| Part of exon 7 | exon7-2 (F) | CACAACGGATTTTGTGAACG |
|  | exon7-2 (R) | AGGAACAAAACCCCTTCTGG |
| Exon 8 | exon8 (F) | CTCTGTATCGGTGGGGTGAC |
|  | exon8 (R) | CAAAAAGCATCGAACACACG |
| Exon 9 | exon9 (F) | TGATCTGCCTGTTCTTTCCA |
|  | exon9 (R) | GGGTTCAGAGGATAGCACCA |
| Promoter (g. 2933–3687) | promoter-1 (F) | TTTACAAAGGAGCACCAGCAGGAGA |
|  | promoter-1 (R) | ATTACATTTAAGAATACTTGTCAGG |
| Promoter (g. 3568–4249) | promoter-2 (F) | AAGTGCCACCCTCCATCC |
|  | promoter-2 (R) | AGCGGTGACAGCCTTTGG |
| Promoter (g. 4110–4744) | promoter-3 (F) | GGTGAGTGTTGTTCTGATTC |
|  | promoter-3 (R) | TCACGTGACTCTCTGGGTCG |
| 3’-UTR (g. 18828–19347) | 3’-UTR-1 (F) | CAACGGCTACTGCAACGAAT |
|  | 3’-UTR-1 (R) | CTCGCTGCCATGAGGATCT |
| 3’-UTR (g. 19272–19787) | 3’-UTR-2 (F) | GAGAAGCCAGAGCCATTCCACCT |
|  | 3’-UTR-2 (R) | GCTCATGCCCCAACAACAACCA |
| 3’-UTR (g. 19717–20407) | 3’-UTR-3 (F) | GCTGCCCTAGAAGTACAATA |
|  | 3’-UTR-3 (R) | GACAGCAACCACAAAGCACAC |
| 3’-UTR (g. 20455–20938) | 3’-UTR-4 (F) | CCCAGAGATAAAGGCTGCCAT |
|  | 3’-UTR-4 (R) | GGAAGCACAGTCTTAATATC |
